# When Intraoperative Hypotension Is Not What It Seems: Systolic Anterior Motion of the Mitral Valve Causing Severe Hypotension During Noncardiac Surgery

**DOI:** 10.1155/cria/4363408

**Published:** 2026-09-21

**Authors:** Aibek E. Mirrakhimov

**Affiliations:** ^1^ Department of Anesthesiology, Perioperative, Critical Care, and Pain Medicine, Division of Adult Cardiothoracic Anesthesiology, University of Kentucky, Lexington, Kentucky, USA, uky.edu

**Keywords:** dynamic left ventricular outflow tract obstruction, intraoperative hypotension, non-cardiac surgery, rescue transoesophageal echocardiography, systolic anterior motion of the mitral valve

## Abstract

Intraoperative hypotension in noncardiac surgery is common and often multifactorial. We report a case of refractory hypotension during orthopedic surgery initially suspected to represent massive pulmonary embolism based on tachycardia and reduced end tidal carbon dioxide. Rescue transesophageal echocardiography demonstrated hyperdynamic biventricular function without right ventricular strain and identified systolic anterior motion of the mitral valve with dynamic left ventricular outflow tract obstruction and posteriorly directed mitral regurgitation. Targeted management with volume expansion, afterload augmentation, and beta blockade resulted in rapid hemodynamic improvement, highlighting systolic anterior motion as an underrecognized and reversible cause of intraoperative hypotension.

## 1. Introduction

Intraoperative hypotension is frequently encountered during noncardiac surgery and has been associated with postoperative organ injury and other adverse outcomes, particularly when sustained or severe [1]. The differential diagnosis is broad and includes hemorrhage, vasodilation, myocardial ischemia, arrhythmia, anaphylaxis, right ventricular failure, and obstructive causes such as pulmonary embolism. Because treatment differs substantially across these mechanisms, persistent or unexplained hypotension requires rapid physiologic clarification rather than repeated empiric escalation.

Rescue transesophageal echocardiography (TEE) provides immediate bedside assessment of ventricular size and function, volume status, valvular pathology, pericardial disease, and major obstructive physiology [2]. This is particularly valuable when clinical features suggest more than one competing diagnosis or when initial resuscitative measures fail.

Systolic anterior motion (SAM) of the mitral valve with dynamic left ventricular outflow tract (LVOT) obstruction is an important but often underrecognized cause of acute perioperative hypotension. Although SAM is classically associated with hypertrophic cardiomyopathy and mitral valve repair, it may also occur in patients without known hypertrophic cardiomyopathy when perioperative conditions create a small, hyperdynamic left ventricle [3–5]. We report a case of refractory intraoperative hypotension initially suspected to represent massive pulmonary embolism, in which rescue TEE identified SAM‐related dynamic LVOT obstruction and redirected management.

## 2. Case Presentation

A 70‐year‐old man with non–small‐cell lung cancer, prior pulmonary embolism treated with apixaban, and hypertension presented for hemiarthroplasty. His preoperative blood pressure was 118/77 mmHg. Approximately 2 h after surgical incision, he developed progressive hypotension with a mean arterial pressure in the mid‐50 s·mmHg and sinus tachycardia up to 126 beats/min. Estimated blood loss was 700 mL, and 2.5 L of crystalloid had been administered. End‐tidal carbon dioxide decreased from the mid‐40 s to the mid‐20 s mmHg.

An additional 500 mL crystalloid bolus did not improve hemodynamics. Arterial blood gas analysis showed pH 7.25, Paco 2 55 mmHg, Pao 2 64 mmHg on Fio 2 1.0, and hematocrit 37%. In the setting of hypotension, tachycardia, hypoxemia, prior pulmonary embolism, and a marked decrease in end‐tidal carbon dioxide, massive pulmonary embolism was considered a leading diagnosis. Epinephrine boluses were administered, vasoactive infusions were prepared, and venoarterial extracorporeal support was activated.

**FIGURE 1:**
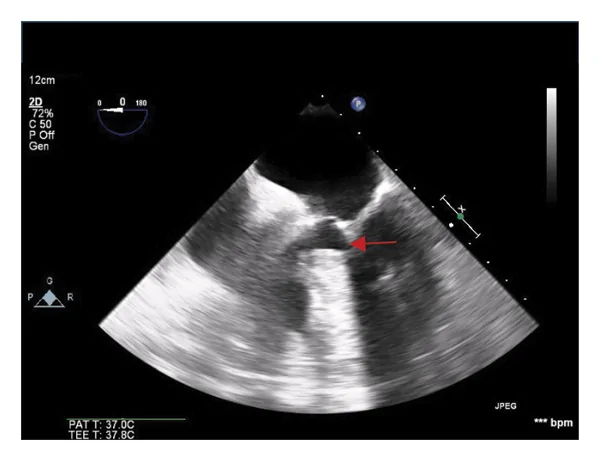
Midesophageal four chamber view in end systole demonstrating SAM of the anterior mitral valve leaflet (red arrow).

**FIGURE 2:**
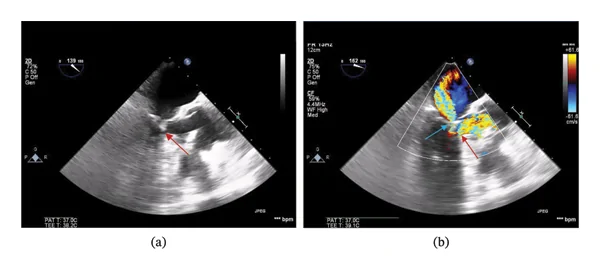
(a) Midesophageal long‐axis view in systole demonstrating SAM of the anterior mitral valve leaflet (red arrow). (b) Midesophageal long‐axis view in systole with color flow Doppler demonstrating flow acceleration through the left ventricular outflow tract (red arrow) and posteriorly directed mitral regurgitation jet secondary to SAM (blue arrow).

**FIGURE 3:**
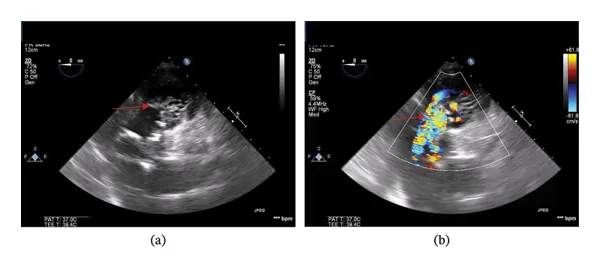
(a) Deep transgastric view demonstrating SAM (red arrow). (b) Deep transgastric view with color Doppler demonstrating flow acceleration through the left ventricular outflow tract (red arrow).

Because hypotension persisted despite volume administration and the mechanism remained uncertain, rescue TEE was performed. TEE demonstrated hyperdynamic biventricular systolic function, a small underfilled left ventricular cavity, and no right ventricular dilation or systolic dysfunction (Figure 1). These findings argued strongly against hemodynamically significant pulmonary embolism at that time. In the midesophageal long‐axis view, the anterior mitral leaflet displaced toward the interventricular septum during systole with near septal contact, consistent with SAM (Figure 2a and Video Clip 1). Color Doppler showed flow acceleration and aliasing in the LVOT, accompanied by a posteriorly directed mitral regurgitation jet caused by impaired leaflet coaptation (Figure 2b and Video Clip 2). The deep transgastric view further supported SAM‐related dynamic LVOT obstruction and provided favorable alignment for LVOT assessment (Figure 3a,b). Unfortunately, because of the urgency of the patient’s intraoperative care, imaging was performed using an older TEE system without functional ECG monitoring. ECG monitoring is important for accurately identifying the phases of the cardiac cycle and should be used whenever available. In its absence, valve motion and ventricular chamber size can provide temporal landmarks. Diastole is characterized by opening of the mitral and tricuspid valves and closure of the aortic and pulmonic valves, whereas systole is characterized by the opposite pattern. End diastole corresponds approximately to the frame immediately before atrioventricular valve closure, when the RV and LV are largest, whereas end systole occurs immediately before atrioventricular valve opening, when the ventricular chambers are smallest.

Management was immediately redirected toward SAM physiology. Additional intravenous volume was administered, afterload was augmented with phenylephrine, and contractility and heart rate were reduced with esmolol. This approach resulted in rapid hemodynamic improvement and a marked reduction in SAM and mitral regurgitation (Video Clip 3). Serial two‐dimensional and color Doppler imaging demonstrated resolution of the flow acceleration and a reduction in the posteriorly directed mitral regurgitation jet. These echocardiographic findings were interpreted alongside improvement in arterial pulse pressure and heart rate, as well as the overall hemodynamic response. LVOT pressure gradients were not measured in this case; however, their assessment would have provided additional quantitative support for these findings.

The patient was extubated to nasal cannula oxygen at the conclusion of surgery and transferred to the postanesthesia care unit (PACU). The patient subsequently experienced an aspiration event requiring reintubation and continued to deteriorate, with worsening oxygenation and increasing ventilatory and vasopressor requirements. After discussion of the prognosis and goals of care, the family elected to transition to comfort‐focused care. The patient died on Postoperative Day 9.

## 3. Discussion

This case illustrates a clinically important diagnostic trap: SAM‐related dynamic LVOT obstruction can mimic other causes of perioperative shock, including pulmonary embolism, hypovolemia, and distributive hypotension. In this patient, prior pulmonary embolism, tachycardia, hypoxemia, hypotension, and a substantial decrease in end‐tidal carbon dioxide made massive pulmonary embolism a reasonable initial concern. However, rescue TEE demonstrated a hyperdynamic, underfilled left ventricle and preserved right ventricular size and function, which redirected the differential diagnosis away from hemodynamically significant pulmonary embolism and toward dynamic LVOT obstruction.

**FIGURE 4:**
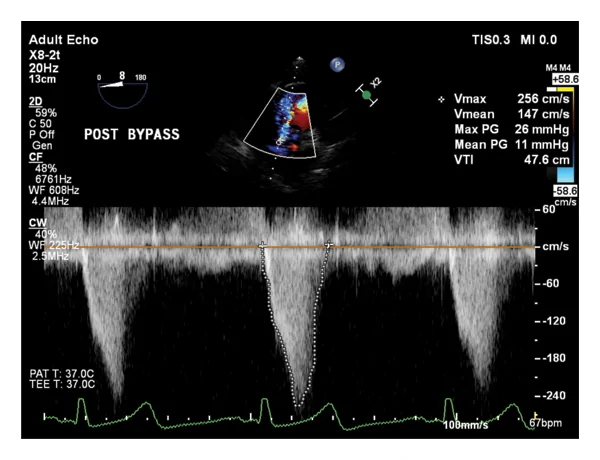
Deep transgastric TEE view with continuous‐wave Doppler across the LVOT and aortic valve, demonstrating a late‐systolic‐peaking, dagger‐shaped spectral envelope in a patient with SAM and dynamic LVOT obstruction.

**FIGURE 5:**
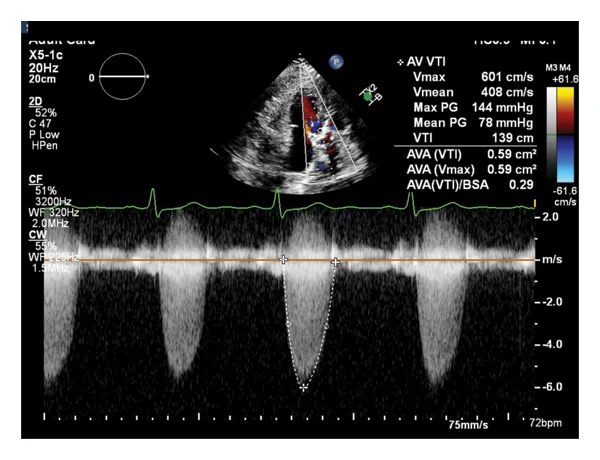
Apical three‐chamber view with continuous‐wave Doppler across the aortic valve, demonstrating the rounded spectral envelope of fixed valvular aortic stenosis and a mean transaortic gradient of 78 mmHg, consistent with severe aortic stenosis.

SAM is a dynamic interaction between mitral valve anatomy, ventricular geometry, and intracavitary flow. Reduced preload and afterload decrease left ventricular cavity size, while tachycardia and increased contractility increase ejection velocity and drag forces on the mitral apparatus. The anterior mitral leaflet may then move into the LVOT during systole, causing dynamic obstruction. Incomplete mitral leaflet coaptation frequently produces mitral regurgitation, which is classically posteriorly directed when anterior leaflet displacement is the dominant mechanism [5]. The continuous‐wave Doppler spectral envelope of dynamic LVOT obstruction typically has a late‐systolic‐peaking, dagger‐shaped contour, as demonstrated in Figure 4. This pattern differs from the broader, more rounded contour generally observed with fixed valvular aortic stenosis, as shown in Figure 5. Figures 4 and 5 were obtained from patients unrelated to the case presented in this manuscript and are included for illustrative comparison.

The management implications are substantial. Empiric inotropes, beta‐agonists, vasodilators, and unnecessary diuresis may worsen SAM by increasing contractility, reducing afterload, or reducing preload. In the absence of primary right ventricular failure, treatment should instead increase left ventricular size and afterload while reducing excessive contractility and tachycardia. Volume expansion, phenylephrine, and short‐acting beta blockade are, therefore, physiologically coherent choices [3, 5]. In this case, the initial use of epinephrine was understandable while massive pulmonary embolism remained a concern; however, after TEE clarified the mechanism, therapy was appropriately redirected.

Echocardiographic diagnosis requires a systematic multiview approach. The midesophageal long‐axis view allows direct visualization of anterior mitral leaflet motion toward the septum. Color Doppler demonstrates LVOT turbulence and may identify the characteristic posteriorly directed mitral regurgitation jet. Continuous‐wave Doppler across the LVOT, when obtainable, typically shows a late‐peaking, dagger‐shaped systolic velocity profile consistent with dynamic obstruction. Transgastric or deep transgastric views may improve Doppler alignment and help quantify the LVOT gradient. A complete examination should also evaluate biventricular function, regional wall motion, pericardial effusion, and alternative causes of shock.

Right ventricular assessment is especially important. SAM may rarely occur secondary to severe right ventricular dilation or failure, in which leftward septal shift and left ventricular underfilling contribute to LVOT narrowing [6, 7]. In that circumstance, isolated beta blockade or excessive volume loading may be harmful, and management should prioritize the underlying right ventricular pathophysiology. Thus, TEE should not merely identify SAM; it should define the broader cardiopulmonary mechanism driving the obstruction.

## 4. Conclusion

SAM‐related dynamic LVOT obstruction is an uncommon but reversible cause of refractory intraoperative hypotension during noncardiac surgery. The clinical picture may resemble pulmonary embolism or other shock states, especially when tachycardia and reduced end‐tidal carbon dioxide are present. Rescue TEE can rapidly identify the correct mechanism and guide counterintuitive but effective management with volume expansion, afterload augmentation, and reduction of heart rate and contractility.

## Funding

No funding was received for this work.

## Consent

Written informed consent for publication of this case and associated images was obtained from the patient’s wife.

## Conflicts of Interest

The author declares no conflicts of interest.

## Supporting Information

Additional supporting information can be found online in the Supporting Information section.

## Supporting information


**Supporting Information 1** Video Clip 1. Midesophageal long‐axis view demonstrating SAM of the anterior mitral valve leaflet.

**Supporting Information 2** Video Clip 2. Midesophageal long‐axis view with color flow Doppler demonstrating flow acceleration through the left ventricular outflow tract and posteriorly directed mitral regurgitation jet secondary to SAM.

**Supporting Information 3** Video Clip 3. Marked reduction in systolic anterior motion of the mitral valve and associated posteriorly directed mitral regurgitation, with corresponding improvement in left ventricular outflow tract flow.

## Data Availability

All data generated during this study are included in this published article.
